# Effect of apneic oxygenation with intubation to reduce severe desaturation and adverse tracheal intubation-associated events in critically ill children

**DOI:** 10.1186/s13054-023-04304-0

**Published:** 2023-01-17

**Authors:** Natalie Napolitano, Lee Polikoff, Lauren Edwards, Keiko M. Tarquinio, Sholeen Nett, Conrad Krawiec, Aileen Kirby, Nina Salfity, David Tellez, Gordon Krahn, Ryan Breuer, Simon J. Parsons, Christopher Page-Goertz, Justine Shults, Vinay Nadkarni, Akira Nishisaki, for National Emergency Airway Registry for Children (NEAR4KIDS) Investigators and Pediatric Acute Lung Injury and Sepsis Investigators (PALISI) Network

**Affiliations:** 1 Respiratory Therapy Department, Children’s Hospital of Philadelphia, Philadelphia, PA USA; 2 Division of Pediatric Critical Care Medicine, The Warren Alpert School of Medicine at Brown University, Providence, RI USA; 3 Division of Critical Care, Department of Pediatrics, Children’s Healthcare of Atlanta, University of Nebraska Medical Center and Children’s Hospital and Medical Center, Omaha, NE USA; 4 Division of Pediatric Critical Care Medicine, Department of Pediatrics, Emory University School of Medicine, Atlanta, GA USA; 5 Division of Pediatric Critical Care, Department of Pediatrics, Dartmouth Hitchcock Medical Center, Lebanon, NH USA; 6 Division of Pediatric Critical Care Medicine, Penn State Health Children’s Hospital, Hershey, PA USA; 7 Division of Pediatric Critical Care Medicine, Department of Pediatrics, Doernbecher Children’s Hospital, Oregon Health and Science University, Portland, OR USA; 8 Department of Critical Care, Phoenix Children’s Hospital, Phoenix, AZ USA; 9 Division of Pediatric Critical Care, University of British Columbia, Vancouver, BC Canada; 10 Division of Pediatric Critical Care, Oishei Children’s Hospital, Buffalo, NY USA; 11 Division of Critical Care, Alberta Children’s Hospital, Calgary, Canada; 12 Division of Critical Care Medicine, Akron Children’s Hospital, Akron, OH USA; 13 Division of Anesthesia and Critical Care Medicine, Children’s Hospital of Philadelphia, Philadelphia, PA USA

**Keywords:** Pediatrics, Tracheal intubation, Airway, Hypoxemia, Apneic oxygenation

## Abstract

**Background:**

Determine if apneic oxygenation (AO) delivered via nasal cannula during the apneic phase of tracheal intubation (TI), reduces adverse TI-associated events (TIAEs) in children.

**Methods:**

AO was implemented across 14 pediatric intensive care units as a quality improvement intervention during 2016–2020. Implementation consisted of an intubation safety checklist, leadership endorsement, local champion, and data feedback to frontline clinicians. Standardized oxygen flow via nasal cannula for AO was as follows: 5 L/min for infants (< 1 year), 10 L/min for young children (1–7 years), and 15 L/min for older children (≥ 8 years). Outcomes were the occurrence of adverse TIAEs (primary) and hypoxemia (SpO_2_ < 80%, secondary).

**Results:**

Of 6549 TIs during the study period, 2554 (39.0%) occurred during the pre-implementation phase and 3995 (61.0%) during post-implementation phase. AO utilization increased from 23 to 68%, *p* < 0.001. AO was utilized less often when intubating infants, those with a primary cardiac diagnosis or difficult airway features, and patient intubated due to respiratory or neurological failure or shock. Conversely, AO was used more often in TIs done for procedures and those assisted by video laryngoscopy. AO utilization was associated with a lower incidence of adverse TIAEs (AO 10.5% vs. without AO 13.5%, *p* < 0.001), aOR 0.75 (95% CI 0.58–0.98, *p* = 0.03) after adjusting for site clustering (primary analysis). However, after further adjusting for patient and provider characteristics (secondary analysis), AO utilization was not independently associated with the occurrence of adverse TIAEs: aOR 0.90, 95% CI 0.72–1.12, *p* = 0.33 and the occurrence of hypoxemia was not different: AO 14.2% versus without AO 15.2%, *p* = 0.43.

**Conclusion:**

While AO use was associated with a lower occurrence of adverse TIAEs in children who required TI in the pediatric ICU after accounting for site-level clustering, this result may be explained by differences in patient, provider, and practice factors.

*Trial Registration* Trial not registered.

**Supplementary Information:**

The online version contains supplementary material available at 10.1186/s13054-023-04304-0.

## Introduction

Tracheal intubation (TI) for critically ill children is a high-risk procedure associated with complications leading to poor intensive care (ICU) outcomes [1–9]. Critically ill children are more likely to have limited physiologic oxygen reserve, hemodynamic instability, and difficult airway anatomical features, in contrast to children intubated in a more controlled setting by anesthesiologists in the operating suites. The occurrence of adverse tracheal intubation-associated events (TIAEs) and peri-intubation hypoxemia have been reported in up to 19% of TIs leading to longer duration of mechanical ventilation, ICU length of stay, and increased mortality [4, 5, 7, 10]. Peri-intubation hypoxemia, in particular, is strongly associated with hemodynamic TIAEs (e.g., cardiac arrest, hypotension, and dysrhythmia) [6, 11, 12]. Risk factors for peri-intubation hypoxemia include age < 1-year, respiratory failure or upper airway obstruction as the indication for TI, less experienced laryngoscopist, and multiple TI attempts [6, 11, 12]. Strategies to reduce peri-intubation hypoxemia may reduce adverse outcomes associated with TI in critically ill children.

Apneic oxygenation (AO) is the application of oxygen flow to the nasopharynx during the apneic phase of TI procedures. The oxygen flow is thought to diffuse to the alveoli and thus extend the safe apnea time during the intubation procedure. AO is shown to be effective in some adult [13–15], pediatric [16–18], and neonatal studies [19].

Our study goal was to implement the AO practice and evaluate the TI safety outcomes across the diverse pediatric ICUs. We hypothesized that implementation of AO practice is feasible and effective in reducing the occurrence of adverse TIAEs and peri-intubation hypoxemia across pediatric ICUs.

## Materials and methods

We implemented AO as a multicenter quasi-experimental, prospective quality improvement intervention across 14 pediatric ICUs. Timing of the implementation varied across the sites. Each ICU’s quality improvement leadership endorsed the intervention. All participating sites were members of the National Emergency Airway Registry for Children (NEAR4KIDS) collaborative and had the infrastructure to collect and timely report TI safety process and outcome data as well as have successfully implemented use of the TI airway safety bundle checklist with maintenance of 80% usage in TIs. Each site’s Institutional Review Board granted approval or exempt status for the ongoing data collection. The Children’s Hospital of Philadelphia served as the Data Coordinating Center (IRB 16–013147).

Inclusion criteria were all primary TIs performed in the participating pediatric ICUs from 2016 to 2020. Secondary TI (i.e., exchange of an existing endotracheal tube for a new tube) were excluded. The first course (defined as initial set of method and approach with a set of medications) of each TI encounter was used for analysis [4, 5]. Study data were collected through the NEAR4KIDS multicenter airway management quality improvement (QI) database. Datapoints included patient, provider, and practice characteristics such as demographics, procedure indication, difficult airway features, primary laryngoscopist discipline and training level, and clinical outcomes. Data were collected, verified, and entered by each site in accordance with previously established operational definitions and the site-specific compliance plan to ensure the capture of > 95% of all TIs. AO use for each intubation was also collected.

### Outcome definitions

The primary outcome was the occurrence of any adverse TIAE, as defined in the NEAR4KIDS operational definitions (Additional file 1: Table A). Secondary outcomes included the occurrence of severe TIAEs, need for multiple attempts, and occurrence of peri-intubation hypoxemia. Multiple attempts were defined as greater than 2 laryngoscopy attempts. Peri-intubation hypoxemia was prospectively defined as oxygen desaturation below 80% for TIs in which patients were able to be pre-oxygenated to at least 90% [20, 21].

### Apneic oxygenation (AO) intervention

A rigorous implementation strategy was adopted, using a specifically developed toolkit which included the following requirements: local quality improvement leadership endorsement, ICU staff education before AO rollout, adoption of universal AO use for all primary TIs, updating TI safety bundle checklist with an AO prompt, and site-level data feedback with adherence data for AO use and adverse outcomes (Additional file 2: Document A). AO was delivered via a standard soft, simple nasal cannula of an age-appropriate size. For better feasibility, oxygen was not heated or humidified. The following oxygen flow rates via nasal cannula were recommended: a minimum of 5 L/min for infants less than 12 months, 10 L/min for children from 1 to 7 years, and 15 L/min for children 8 years and older. These guidelines were based on multicenter NEAR4KIDS consensus and previous adult evidence [13]. Placement of the nasal cannula was advised to be performed either before induction or soon after induction in accordance with the child’s tolerance of the nasal cannula. If a child was receiving heated humidified high-flow oxygen therapy with a flow at or greater than the flow recommended for the age category, we endorsed the use of the same flow rate through the system already in place, with an increase in the fraction of inspired oxygen (FiO_2_) to 1.00. If the child was being treated with noninvasive positive pressure ventilation, we advised placement of the nasal cannula at the time of induction or when the providers began manual bag-mask ventilation.

### Statistical analyses

For summary statistics, categorical variables were described as a number and percent and evaluated with Chi-square or Fisher’s exact test. Non-normally distributed continuous variables were reported as median and interquartile ranges (IQR) and evaluated using Wilcoxon rank-sum test. All statistical analysis was performed using Stata 15.1 (Stata Corp, College Station, TX, USA). A two-sided *p* value of less than 0.05 was considered statistically significant.

In our *primary analysis*, we evaluated the effectiveness of the AO intervention, comparing the rate of adverse TIAEs among children who received TI with AO versus without AO, from January 2016 to December 2020. As our data were clustered by site (i.e., multiple TIs per site), we constructed a hierarchal data structure and analyzed the data using a generalized estimating equations (GEE) logistic regression model using an independent correlation structure. We similarly performed the analyses for the secondary outcomes.

In the *secondary analysis*, we also adjusted for subject-level covariates. This process included the identification of candidate covariates based on a priori knowledge related to the patient, provider, and practice characteristics on AO use and the primary outcome. Conservatively, we also included the covariates with the association with AO use at *p* < 0.1 level. We also conducted subgroup analyses to identify heterogeneity in the effect of AO on the primary outcome. *p* < 0.1 was considered as significant for heterogeneity.

In sensitivity analyses, we compared the primary outcome between the pre- and post-AO implementation across the participating site to evaluate the effect of AO implementation practice. Like the primary analysis, we accounted for the site-level clustering by GEE.

### Sample size estimation

Based on the ongoing NEAR4KIDS TI registry, every month approximately 200 TIs were reported from 28 sites at the time of study inception. With the baseline rate of adverse TIAE occurrence at 15%, with 20% relative reduction (3% absolute reduction), a total of 2280 TIs was required. Due to initial uncertainties in the number of participating sites with the QI intervention, we aimed to recruit at least ten ICUs throughout the study period.

## Results

**Fig. 1:**
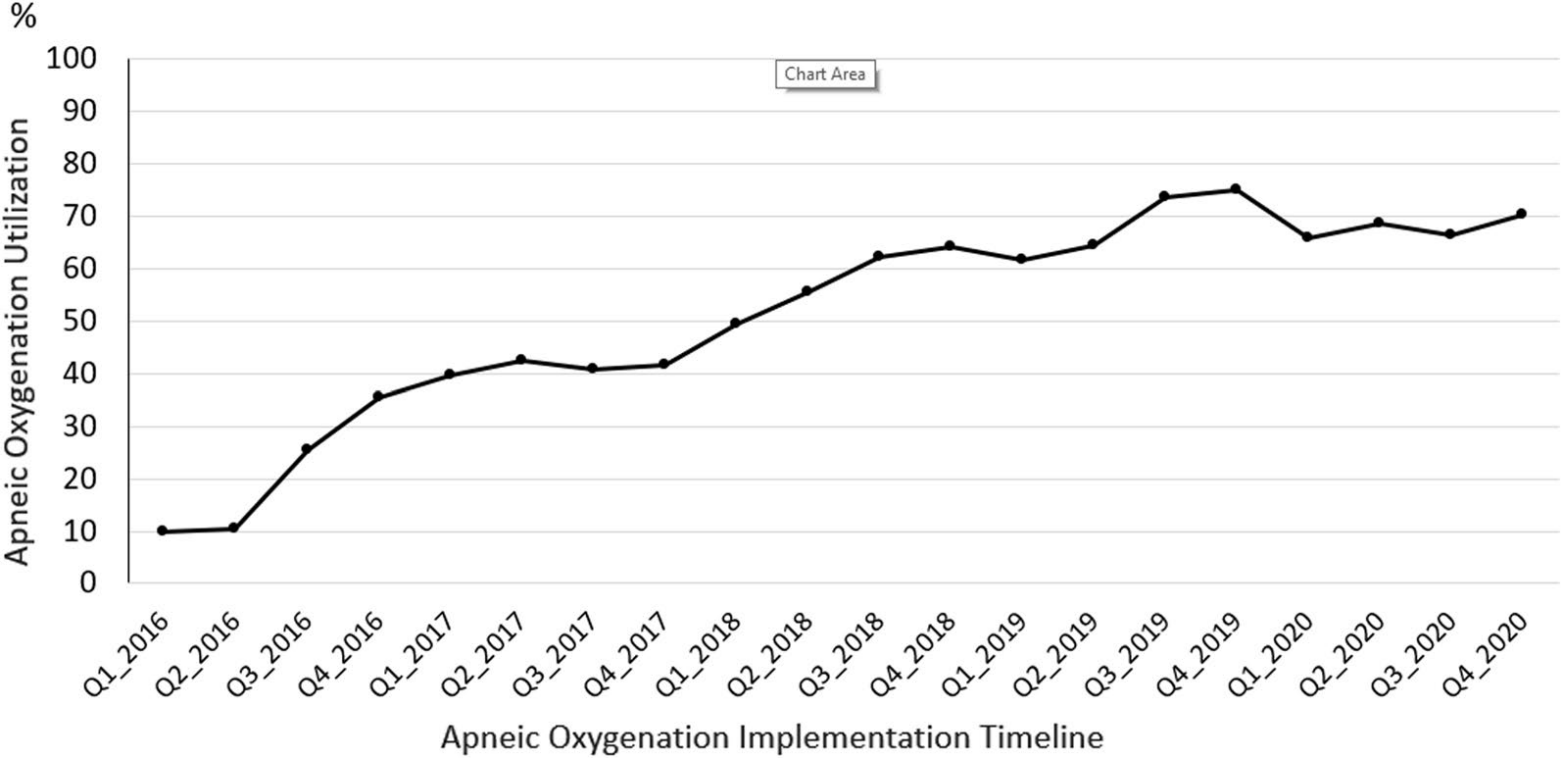
Apneic Oxygenation Use over time (*n* = 6549)

A total of 14 pediatric ICUs implemented AO practice between 2016 and 2020. AO utilization increased after formal AO implementation: 587/2554 (23%) to 2731/3995 (68%), *p* < 0.001. (Fig. 1).

The AO utilization uptake was variable across the sites after the implementation. (Additional file 3: Figure A).

### Apneic oxygenation utilization

AO was utilized more often in older children and in those with procedural indications for intubation. It was also used more often in TIs performed by nurse practitioners and those utilizing video laryngoscopy. Conversely, AO was used less often in TIs in infants, those with a primary cardiac diagnosis or difficult airway features, patients intubated due to respiratory or neurological failure or shock, and those using a direct laryngoscope. (Table 1).

**Table 1:** Patient Characteristics

Patient characteristic	With apneic oxygenation n = 3318	Without apneic oxygenation n = 3231	*p* value
Age category			< 0.001
Infant (< 12 month)	1,174 (35.4%)	1,349 (41.8%)	
Young child (1–7 year)	1,109 (30.5%)	985 (30.5%)	
Older child (8–17 year)	863 (26.0%)	761 (23.6%)	
Adult (18 year or older)	172 (5.2%)	136 (4.2%)	
Weight (kg, median, IQR)	13 (6.5–33)	10.9 (5.4–28)	
Diagnosis			< 0.001
Respiratory	1529 (46.1%)	1624 (50.3%)	
Cardiac	108 (3.3%)	318 (9.8%)	
Neurological	971 (29.3%)	656 (20.3%)	
Shock	380 (11.5%)	299 (9.3%)	
Trauma/Traumatic brain injury	90 (2.7%)	95 (2.9%)	
Other	240 (7.2%)	239 (7.4%)	
Indication for intubation			
Respiratory failure	2002 (60.3%)	2162 (66.9%)	< 0.001
Shock	339 (10.2%)	421 (13.0%)	< 0.001
Neurological	224 (6.8%)	360 (11.1%)	< 0.001
Procedural	819 (24.7%)	429 (13.3%)	< 0.001
Difficult airway			
History of difficult airway	457 (13.8%)	501 (15.5%)	
Difficult airway feature	796 (24.0%)	924 (28.6%)	
Airway clinician			< 0.001
Attending	402 (12.2%)	465 (14.4%)	
Fellow	1818 (54.9%)	1722 (53.3%)	
Resident	377 (11.4%)	334 (10.3%)	
Nurse Practitioner	383 (11.6%)	283 (8.8%)	
Hospitalist	64 (1.9%)	27 (0.8%)	
Respiratory Therapist	65 (2.0%)	30 (0.9%)	
Subspecialist/Physician assistant/Other	200 (6.0%)	369 (11.4%)	
Vagolytic	1247 (37.6%)	751 (23.2%)	< 0.001
Ketamine	1647 (49.6%)	1240 (38.4%)	< 0.001
Propofol	632 (19.1%)	378 (11.7%)	< 0.001
Neuromuscular blockade	3,177 (95.8%)	2961 (91.6%)	< 0.001
Device			
Direct laryngoscope	1185 (35.7%)	2062 (63.9%)	< 0.001
Video laryngoscope	2109 (63.6%)	1127 (34.9%)	
Other device	24 (0.7%)	42 (1.2%)	

### Primary outcome

In a univariate analysis, AO use was associated with a lower adverse TIAE rate; with AO 349/3318 (10.5%) versus without AO 436/3231 (13.5%), absolute difference: 3.0% (95% CI 1.4–4.5%), *p* < 0.001 (Table 2). In the *primary analysis* with a GEE logistic regression to account for site clustering, AO use was significantly associated with lower occurrence of adverse TIAEs: OR 0.75, 95% CI 0.58–0.98, *p* = 0.032. In the *secondary analysis* with a multivariable logistic regression model, AO use was not associated with a lower adverse TIAE rate: adjusted OR 0.90, 95% CI 0.72–1.12, *p* = 0.334 after controlling for patient age, TI indication (respiratory failure, neurological failure, shock, and procedure), difficult airway history and clinical features, medication (vagolytic, ketamine, propofol, and neuromuscular blockade), provider training level and video laryngoscope use, and site clustering (Table 3).

**Table 2:** Univariate analyses: adverse tracheal intubation-associated events, severe tracheal intubation-associated events, and peri-intubation hypoxemia (SpO_2_ < 80%) in tracheal intubations with or without apneic oxygenation

Outcome	With apneic oxygenation (%)	Without apneic oxygenation (%)	Unadjusted Odds Ratio (95% CI)	*p* value
Any adverse TIAE (primary outcome)	10.5	13.5	0.75 (0.58–0.98)	0.032
Severe TIAE	4.0	5.8	0.67 (0.48–0.94)	0.021
Multiple attempts (> 2 attempt)	8.4	9.5	0.87 (0.70–1.08)	0.214
Severe hypoxemia (SpO_2_ < 80%)	14.9	16.3	0.90 (0.66–1.23)	0.502

Unadjusted odds ratio was calculated with logistic regression with generalized estimating equations.

*CI* denotes confidence interval

**Table 3:** Multivariable analysis (*secondary analysis*): the occurrence of adverse tracheal intubation-associated events in patients receiving tracheal intubations with or without apneic oxygenation

Variable	Adjusted odds ratio	95% confidence interval	*p* value
Apneic oxygenation use	0.90	0.72–1.12	0.334
Age			
Infant	Reference	NA	NA
Young child (1–7 year)	1.09	0.89–1.34	0.384
Older child (8–17 year)	0.99	0.74–1.33	0.934
Adult (18 year or older)	1.13	0.77–1.67	0.521
Indication for intubation			
Respiratory failure	1.24	1.05–1.46	0.010
Shock	1.60	1.30–1.97	< 0.001
Neurological	0.99	0.75–1.33	0.967
Procedural	0.81	0.64–1.03	0.084
History of difficult airway	0.82	0.66–1.02	0.080
Difficult airway feature	1.24	1.05–1.47	0.013
Clinician			
Attending	Reference	NA	NA
Fellow	0.76	0.59–0.97	< 0.001
Resident	1.12	0.79–1.57	0.521
Nurse practitioner	0.85	0.59–1.22	0.371
Hospitalist	0.94	0.65–1.35	0.729
Respiratory therapist	1.85	1.06–3.26	0.032
Subspecialist/Physician assistant/Other	0.84	0.61–1.18	0.317
Vagolytic	1.60	1.11–2.31	0.012
Ketamine	0.81	0.64–1.04	0.096
Propofol	0.65	0.41–1.01	0.055
Neuromuscular blockade	0.75	0.52–1.08	0.120
Direct laryngoscope	Reference	NA	NA
Video laryngoscope	0.61	0.49–0.76	< 0.001

*NA* not applicable

### Secondary outcomes

In univariate analysis, AO use was associated with lower severe TIAEs (with AO 4.0% vs. without AO 5.8%, *p* = 0.001) but not with multiple TI attempts (with AO 8.4% vs. without AO 9.5%, *p* = 0.111) or severe hypoxemia (with AO 14.9% vs. without AO 16.3%, *p* = 0.113). After accounting for site clustering, AO use was associated with lower severe TIAEs (OR 0.67, 95% CI 0.48–0.94, *p* = 0.021) but not associated with lower multiple attempts (OR 0.87: 95% CI 0.70–1.08, *p* = 0.214) or severe hypoxemia (OR 0.90: 95% CI 0.66–1.23, *p* = 0.502).

### Subgroup analysis

**Fig. 2:**
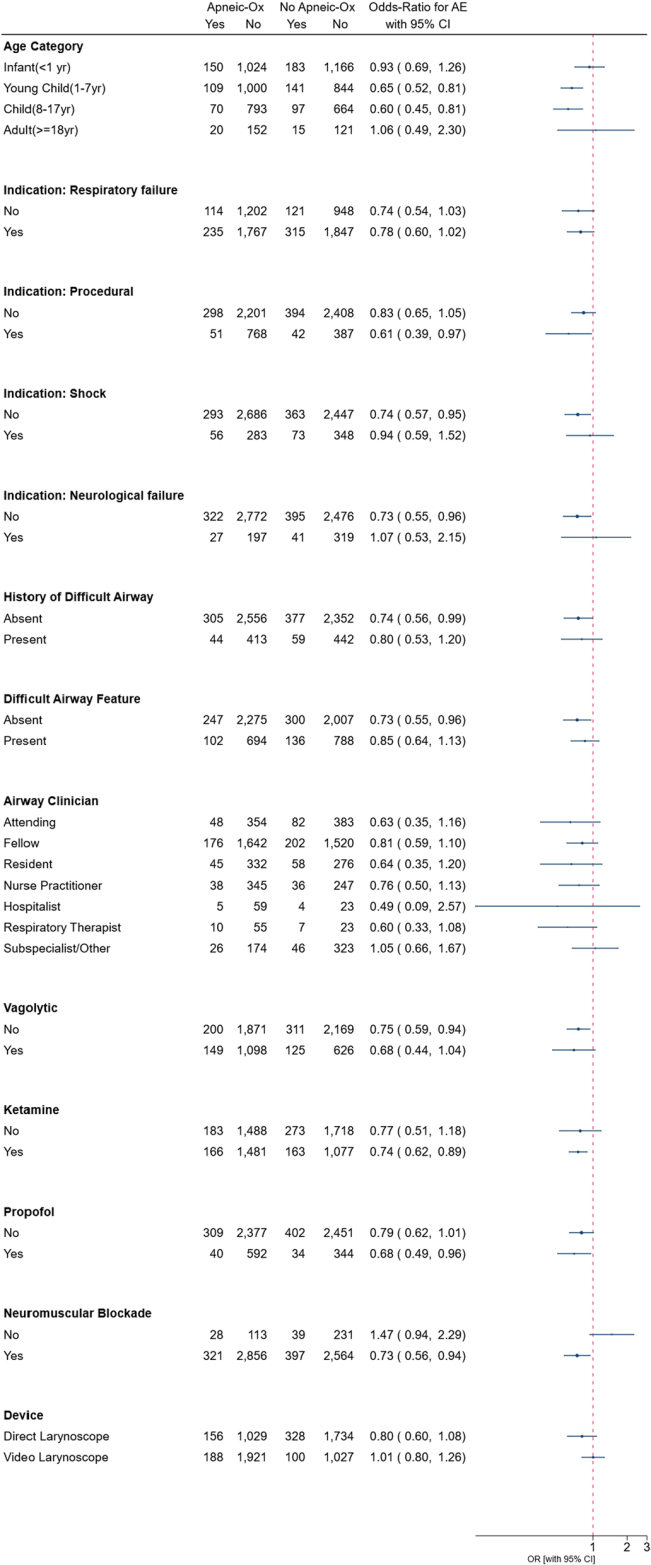
Forrest plot for subgroup analysis test for homogeneity

For each age subgroup, the association between AO use and the primary outcome varied: infants OR 0.93, 95% CI 0.74–1.18, young children (1–7 years) OR 0.65, 95% CI 0.50–0.85, older children (8–17 years) OR 0.60, 95% CI 0.44–0.83, and adults (≥ 18 years) OR 1.06, 95% CI 0.53–2.14, *p* = 0.070 for test of homogeneity (Fig. 2). There was heterogeneity in the association between AO use and the primary outcome among patients who received neuromuscular blockade versus those that did not: with neuromuscular blockade: OR 0.73, 95% CI 0.62–0.85, without neuromuscular blockade: OR 1.47, 95% CI 0.86–2.50, test of homogeneity = 0.013. The association between AO use and the primary outcome was consistent across other subgroups.

### Sensitivity analysis

Compared to pre-AO implementation, post-AO implementation was not associated with lower adverse TIAE rates; pre-implementation 324/2554 (12.7%) vs. post-implementation 461/3995 (11.5%), *p* = 0.164 (Additional file 4: Table B). After accounting for site clustering by GEE, post-implementation was not associated with lower adverse TIAE rates: OR 0.90, 95% CI 0.55–1.45, *p* = 0.662. Similarly, post-implementation was not associated with severe TIAEs, multiple attempts, or severe hypoxemia. These results were also similar with stratified analyses based on site-level compliance (sites those achieved at least 80% AO use vs. sites those did not).

## Discussion

Within the armamentarium of the pediatric intensivist, there are few interventions that may mitigate physiologic disturbances and optimize physiologic state prior to TI. Previous work from this consortium has identified peri-intubation hypoxemia as a potentially modifiable risk factor associated with adverse TIAEs [20]. Extrapolating from experiences in the operating room, AO was postulated to improve hypoxemia during the apneic phase of laryngoscopy and to reduce physiologic derangements during TI. To our knowledge, this is the first multicenter study assessing the effectiveness of AO in critically ill children across different ages and clinical conditions undergoing TI in diverse ICU settings. Our data show that AO utilization increased among participating sites following a focused implementation as a QI intervention. AO was utilized more often in older children and with procedural indication for TI and less often in the TIs indicated for respiratory failure, those with difficult airway features, and with direct laryngoscope. Moreover, in the primary analyses accounting for clustering by sites, AO use was associated with lower adverse TIAE and severe TIAE rate. However, AO use was not associated with reduction in either multiple attempts or severe peri-intubation hypoxemia. AO use was also no longer significantly associated with lower adverse TIAEs after adjusting for patient, provider, and practice level covariates in the *secondary analysis*.

Our study findings were consistent with recent studies in neonatal ICU and pediatric operating rooms. A randomized control trial by Hodgson et al. [19] demonstrated that nasal high-flow therapy during TI procedures improved the likelihood of successful intubation on the first attempt without physiological instability in the premature infant (median 27.9 weeks) in two Australian neonatal ICUs. The success rate in the intervention group was 50% whereas the control group was 31.5%, with adjusted risk difference 17.6% (95% CI: 6–29.2). Notably, their intervention included 8 L/min of air flow with varying FiO_2_ based on pre-intubation support.

Soneru et al. evaluated the clinical effectiveness of AO in children in the operating room. In their randomized trial, they applied 5 L/min in children < 8 years who received laryngoscopy by trainees. The proportion of hypoxemia (SpO_2_ ≤ 90%) was 4% in the intervention group and 31% in the control group (relative risk 0.14, 95% CI 0.07–0.30). The treatment effect of AO was similar in the infant subgroup (relative risk 0.11, 9% CI 0.03–0.33).

In our single center study at a large pediatric ICU, the implementation of AO was effective in reducing moderate hypoxemia (SpO_2_ < 80%) from 15.4 to 11.8%, *p* = 0.049. This effect remained significant (adjusted OR: 0.55, 95% CI 0.34–0.88) after accounting for TI indications and video laryngoscope use [22]. While the reduction in hemodynamic derangement (i.e., hypotension, dysrhythmia, and cardiac arrest) did not reach statistical significance (3.2–2.0%, *p* = 0.155), this was deemed to be underpowered in this study. These study findings were supportive for the use of AO in reducing peri-intubation hypoxemia and severe TIAEs. This was consistent with our study results which demonstrated a reduction in all TIAEs and severe TIAEs, in which severe physiological derangement was captured.

In the secondary analysis of our study, the occurrence of adverse TIAEs, however, was no longer significantly different after adjusting for patient, provider, and practice differences between the patients who received AO and those who did not. This result could be explained by several factors: (1) AO was preferentially used in the lower risk patients or (2) AO use may be a surrogate for overall higher quality of advanced airway management. Despite this, the direction of odds ratios in all adverse TIAEs, severe TIAEs, peri-intubation hypoxemia, and TI with multiple attempts in both primary and secondary analyses seemed to justify the routine use of AO in TIs for critically ill children.

It is notable that our study result showed heterogeneity of AO’s treatment effect in reducing adverse TIAEs across the age groups. The effect was the largest in young children (1–7 years), while it was not effective in adult patients (≥ 18 years). It is possible that 15 L/min of oxygen via nasal cannula for an adult size patient may be insufficient in extending the safe apneic window for laryngoscopy, as suggested by adult studies [23]. It is possible that the effectiveness of apneic oxygenation was diminished in our study with the use of relatively low-flow oxygen compared to studies utilizing high-flow oxygen, including a series of trans-nasal humidified rapid insufflation ventilatory exchange (THRIVE) studies [16]. A multicenter randomized trial using high-flow oxygen is ongoing for emergency intubation in critically ill children in Australia and New Zealand [24].

Humphrey et al. [17] demonstrated in a randomized control trial that healthy children receiving trans-nasal humidified high-flow oxygen at 60 L/min had a longer safe apnea time without an effect in carbon dioxide clearance. Intriguingly, a study by Riva et al. [16] showed that healthy pediatric patients (1–6 years) who received high-flow 100% oxygen (2 L/kg/min) via nasal cannulas did not have longer safe apnea time compared with low-flow oxygen (0.2 L/kg/min). We chose the use of routine soft nasal cannula as opposed to a rigid, thick cannula required for high-flow nasal cannula therapy to optimize mask seal during bag-mask ventilation with the nasal cannula. From the practical standpoint, the use of a standard oxygen flow system as opposed to setting up a humidified high-flow system was more feasible, economical, and practical in the ICU. However, critically ill children may have diminished oxygen reserve, similar to critically ill adult patients. Adult ICU studies that examined 15 L/min of oxygen via nasal cannula or high-flow humidified oxygen both failed to show a reduction in hypoxemic events in contrast to studies in the emergency department [25–28].

It is notable that AO was utilized more often in older children and in children with procedural indications. In addition, AO was used less often in TIs indicated for respiratory failure, with difficult airway features, and with direct laryngoscope. Older patients with larger functional residual capacity may tolerate longer apneic times compared to younger patients. This may confer some degree of selection bias, possibly explaining the positive effect of AO seen in the univariate analyses for both primary and secondary outcomes.

There was no significant difference in the adverse TIAE or peri-intubation hypoxemia between before and after AO implementation periods across the sites, while the primary analysis showed the use of AO was associated with lower adverse TIAE and severe TIAE. This nonsignificant result in before- and after-comparisons may be explained by the relatively low AO practice adherence (68% during post-implementation) as well as variability across the site shown in the supplemental figure. Prior work by Davis et al. [29] identified through focus group that barriers to AO implementation included device accessibility, delay in patient care, insufficient staff education, presence of nasal cannula that potentially impairs mask seal during bag-mask ventilation, and limited clinical evidence to support AO use. To address these concerns, we utilized our practical solution from the pilot center experience. These include additional staff education using video demonstrations, standard use of two-person bag-mask ventilation to improve mask seal, and the use of a soft nasal cannula.

We recognize several limitations to this study. First, this study was conducted as a quality improvement project involving various pediatric ICUs, rather than a prospective, randomized control trial. Thus, the timing of rollout or allocation of intervention was not randomized. As discussed above, the study result is subject to selection bias. Uptake of AO practice was variable across the sites. It is likely that the knowledge sharing across the NEAR4KIDS supported the uptake of AO practice in the late-coming sites. Second, the heterogeneity of the patient cohort and variation in provider and staff facility with utilizing AO, concurrent with possible perceptions of ineffective outcomes, may have led to selection bias among the sites in whom AO was utilized. AO was used more often in TIs performed by nurse practitioners and with the video laryngoscope, whereas AO was used less often in TIs with the indication of respiratory failure, difficult airway features, and the use of direct laryngoscopy. Third, we did not capture the time to desaturation because this was a pragmatic quality improvement study. It has been postulated that AO may be most beneficial when utilized in patients with difficult airway, or in intubations where the primary laryngoscopist is a trainee [30]. However, we did not observe effect modification in either the difficult airway population or the laryngoscopist’s discipline or training level.

## Conclusions

While AO use as a quality improvement intervention was associated with a lower occurrence of adverse TIAEs and severe TIAEs in critically ill children who required TI in the pediatric ICU, this result may be explained by differences in patient, provider, and practice factors who received AO versus those who did not.

## Supplementary Information

**Additional file 1. Supplemental Table A.** Adverse Tracheal Intubation Associated Events, Severe vs Non-Severe Events Defined by the National Emergency Airway Registry for Children (NEAR4KIDS) Operational Definitions.

**Additional file 2. Supplemental Document A.** Apneic Oxygenation Implementation Toolkit.

**Additional file 3. Supplemental Figure A.** Apneic Oxygenation (AO) Use (%) before and after implementation of the intervention across the sites.

**Additional file 4. Supplemental Table B.** Multivariable analysis: The Occurrence of Adverse Tracheal Intubation Associated Events in Patients who received Tracheal Intubations Before and After Apneic Oxygenation Implementation

## Data Availability

Data are available upon request from authors.

## Abbreviations

TI: Tracheal intubation
ICU: Intensive care unit
TIAEs: Tracheal intubation-associated events
AO: Apneic oxygenation
NER4KIDS: National Emergency Airway Registry for Children
QI: Quality improvement
SpO2: Oxygen saturation
FiO2: Fraction of inspired oxygen
IQR: Interquartile range
GEE: Generalized estimating equations
