# Supplementary material for: Effect of apneic oxygenation with intubation to reduce severe desaturation and adverse tracheal intubation-associated events in critically ill children
Source: Crit Care. 2023 Jan 17;27:26. doi: 10.1186/s13054-023-04304-0 (PMC9847056; doi:10.1186/s13054-023-04304-0)
Supplement: Supplementary file 1 — Additional file 1. Supplemental Table A. Adverse Tracheal Intubation Associated Events, Severe vs Non-Severe Events Defined by the National Emergency Airway Registry for Children (NEAR4KIDS) Operational Definitions. [file 13054_2023_4304_MOESM1_ESM.docx]

Supplemental Table A. Adverse Tracheal Intubation Associated Events, Severe vs Non-Severe Events Defined by the National Emergency Airway Registry for Children (NEAR4KIDS) Operational Definitions

| **Severe TIAE** | **Non-severe TIAE** |
| --- | --- |
| Cardiac arrest without ROSC | Esophageal intubation immediate recognition |
| Cardiac arrest with ROSC | Main stem bronchial intubation |
| Esophageal intubation delayed recognition | Emesis without aspiration |
| Emesis with aspiration | Hypertension requiring intervention |
| Hypotension requiring intervention | Epistaxis |
| Laryngospasm | Lip trauma |
| Malignant hyperthermia | Medication Error |
| Pneumothorax/pneumo-mediastinum | Dysrhythmia |
| Direct airway injury | Pain/agitation |
| Gum/dental trauma |  |

ROSC denotes return of spontaneous circulation.
