# Supplementary material for: Effect of apneic oxygenation with intubation to reduce severe desaturation and adverse tracheal intubation-associated events in critically ill children
Source: Crit Care. 2023 Jan 17;27:26. doi: 10.1186/s13054-023-04304-0 (PMC9847056; doi:10.1186/s13054-023-04304-0)
Supplement: Supplementary file 2 — Additional file 2. Supplemental Document A. Apneic Oxygenation Implementation Toolkit. [file 13054_2023_4304_MOESM2_ESM.pdf]

Supplemental Document A – Implementation Toolkit

Airway Bundle QI Project: Apneic oxygenation

Congratulations! Your site is selected to be one of the vanguard sites for the next Quality Improvement (QI) Project for the NEAR4Kids project, **apneic oxygenation**. We have learned many lessons from the Airway bundle checklist implementation that will hopefully make your process a smooth transition with success.

It is important to know that the NEAR4Kids project is a QI project and needs to be rolled out just as you do for all other QI safety bundles (i.e. VAP, CLABSI, CAUBI, sedation pathway). You will need to show that your unit has a commitment to the project by providing a letter from your QI chair or unit medical director (template attached). We also recommend that you continue to work with **a multidisciplinary team** to assist with education, implementation and continued maintenance to ingrain this safety QI into the culture of your unit. The multidisciplinary team should include MD (attending, fellow), RN, RT, NP, PA &/or research assistant depending on the make-up of the providers in your unit. Each discipline will have a varied level of participation depending on the commitment they can make. At a minimum it is best to have a point person that can give input on workflow and ensure education and information is translated to their own teams.

Apneic oxygenation <u>is best utilized for any intubations in your ICU</u>, because our data showed desaturation occurs commonly even patients without lung pathology. The occurrence of desaturation is associated with increased adverse tracheal intubation associated events rate. When correctly used, apneic oxygenation can reduce risk of desaturation and may decrease adverse events rate. However, the use of apneic oxygenation requires staff education and pre-planned approach.

We recommend that you develop inclusive education to be provided at least 2 weeks prior to implementation so that everyone knows what will be happening, where to get everything, and the purpose of this QI project. You may choose to include simulation to demonstrate how it works. We have included a copy of the online education used from CHOP. There will be also a demonstration video on the NEAR4Kids website soon. Feel free to include this in your education. It is also helpful to provide compliance feedback to disciplines so they can assist with achieving the team goal of 80% compliance for both at risk and intubated patients.

Lastly, we recommend that you continue to report the compliance rates and trend in TIAE rates at your quality improvement committee.

Please let us know if there are questions or barriers to implementation that we can assist you with.

Sincerely,

Akira Nishisaki

Natalie Napolitano

Hayley Buffman

Checklist for QI Implementation:

- ☐ Receive letter of support and send to Hayley Buffman
- ☐ Form Multidisciplinary Airway QI Team
- ☐ Update checklist for apneic oxygenation and send to QI committee for approval
- ☐ Determine work flow that best suits your unit culture including who has ultimate ownership
- ☐ Outline compliance monitoring plan
- ☐ Set Go Live date and notify us
- ☐ Develop education plan for each discipline
- ☐ Go Live

[Sample letter]

Dear National Emergency Airway Registry for Children (NEAR4KIDS) Quality Improvement Committee,

We reviewed and discussed the introduction of apneic oxygenation as the NEAR4KIDS (National Emergency Airway Registry for Children) quality improvement project. This letter confirms our endorsement of implementation of apneic oxygenation as the quality improvement project in our pediatric ICU. Our scheduled starting date for implementation of the apneic oxygenation is XXXXXXX (date).

Pediatric ICU Quality improvement committee Chair

Date:

____ _____________________________

Airway Bundle Checklist (FRONT and BACK)

Date: _______________ **[Place patient sticker/stamp here]**

Time: _______________

Front page completed (check all that apply):

☐ On admission ☐ During/after rounds ☐ Prior to intubation ☐ After intubation

☐ Prior to extubation By ____

Assessment for ANTICIPATED Airway Management

Intubation Risk Assessment

**Difficult Airway**	**History** of difficult airway?	YES	NO
	**Physical**? (e.g. small mouth, small jaw, large tongue, or short neck)	YES	NO
**At Risk For:**	High risk for rapid **desaturation** during intubation	YES	NO
	Increased ICP, pulmonary hypertension, need to avoid **hypercarbia**	YES	NO
	**Unstable hemodynamics** (e.g. hypovolemia, potential need for fluid bolus, vasopressor, CPR)	YES	NO
	Other risk factors? ______________________________	YES	NO

Planning (all risk noted above should be considered in plan)

Who will intubate? (Specify primary provider who will perform **first** laryngoscopy):

☐ Resident ☐ Fellow ☐ NP ☐ Attending ☐ Anesthesiologist ☐ ENT physician ☐ RT ☐Other - Specify below:

____

How will we intubate? **Method:** ☐ oral vs. ☐ nasal **ETT Type:** ☐ Cuffed ☐ Uncuffed

ETT Size: ☐ 3.0 ☐ 3.5 ☐ 4.0 ☐ 4.5 ☐ 5.0 ☐ 5.5 ☐ 6.0 ☐ 6.5 ☐ 7.0 ☐ 7.5 ☐ 8.0 ☐Other: _____

Device: ☐ Laryngoscope ☐ LMA ______ ☐ Glidescope ______ ☐CMAC ☐Other: _____________

Blade: ☐ Mac _______ ☐ Miller _________ ☐ Wis-Hipple ________

Meds: ☐ Atropine_____mg ☐ Glycopyrrolate_____mcg

☐ Fentanyl_____mcg ☐ Midazolam_____mg ☐ Ketamine_____mg ☐ Propofol______mg

☐ Rocuronium______mg ☐ Vecuronium_____mg

Apneic Oxygenation: YES / NO______L/min (<1y = 5L; 1-7y = 10L; $\geq$8y = 15L)

Other: ____

When will we intubate? (Describe the timing of airway management):

☐ Prior to procedure at:________ ☐ Mental Status Changes ☐ Hypoxemia refractory to CPAP: SpO2 <______%

☐ Ventilation failure refractory to NIV ☐ Loss of Airway Protection ☐Other: ______________________________

____

Backup? Advanced Airway Provider: ☐ Attending ☐ Anesthesia ☐ ENT ☐ Fellow ☐Other: ____________

☐ Difficult Airway Cart ☐ Difficult Airway Emergency Page ☐ Other:________________________________

____

NOT PART OF THE MEDICAL RECORD

Immediate Pre-Intubation Procedure TIME OUT

Date:_____________
Time: _____________

☐ **Front page not filled out: Why?** ____

(Complete immediately before intubation)

☐ **Right Patient**: Confirm 2 identifiers and allergy status.<br>Did the patient eat or drink in the last 6 hours?
☐ **Right Plan**: Review and revise the FRONT PAGE plan
☐ **Right Prep**: Patient accessible and positioned correctly, bed cleared for intubation, working IV?
☐ **Right Equipment**: SOAP (e.g Suction, Oxygen, Airway, Personnel), IV fluid bolus readily available?
☐ **Right Monitoring**: BP cycling frequently, different extremity from pulse ox, pulse ox volume?
☐ **Right Rescue plan**: Difficult Airway cart/kit and equipment available? Who can we call for assistance?<br>***Double press ASCOM Alert Button, or call Emergency# 4CODE***<br>***State: "Stat Airway Emergency" (Provide Location)***
☐ **Right Attitude**: State out loud:<br>**"IF anybody has a concern at any time during the procedure please SPEAK UP."**

☐ **Designate who will press record if CMAC is in use.**

Other PATIENT SPECIFIC preparation:

Post-Procedure TIME OUT

□ **All team members performed well without technical/communication challenges.**

Or

Briefly describe below (comments by provider)

	What did we do well?	What can we improve upon?
1. RT		
2. Nurse		
3. Resident		
4. NP/PA		
5. Fellow		
6. Attending		

ETT Cuff adjusted to minimal leak: YES / NO **Goal SBS_______(- 3 to +2)**

Was the patient difficult to ventilate? YES/NO **Was the Patient difficult to Intubate? YES/NO**

****If Yes to either question please remember to put an ALERT in Epic and a SIGN at the bedside.****

☐ **NEAR4Kids data form completed after intubation?**

Here is the link to download the video file directly:
https://player.vimeo.com/external/181693239.hd.mp4?s=6be3bb14bc7c3e2ad9bb017fcbf610746c1fc587&profile_id=174&download=1

You may also access the video online (as well as download it from this review page):
https://vimeo.com/user8989405/review/181693239/82898c940d

Mark Sim

Jane Sim

Airway Bundle Checklist (FRONT and BACK)

Date: 5/24/2016 [Place patient sticker/stamp here]
Time: 15:00 Sim, Mark
MRN: 12345678

Front page completed (check all that apply):
☑ On admission ☐ During rounds ☐ After Rounds ☐ Just prior to intubation ☐ Prior to Extubation

By Nishisaki

Assessment for ANTICIPATED Airway Management

Intubation Risk Assessment

**Difficult Airway**	**History** of difficult airway?	YES	(NO)
	**Physical**? (e.g. small mouth, small jaw, large tongue, or short neck)	YES	(NO)
**At Risk For:**	High risk for rapid **desaturation** during intubation	(YES)	NO
	Increased ICP, pulmonary hypertension, need to avoid **hypercarbia**	YES	(NO)
	**Unstable hemodynamics** (e.g. hypovolemia, potential need for fluid bolus, vasopressor, CPR)	YES	(NO)
	Other risk factors? ________	YES	(NO)

Planning (all risk noted above should be considered in plan)

Who will intubate? (Specify primary provider who will perform **first** laryngoscopy):
☐ Resident ☑ Fellow ☐ NP ☑ Attending ☐ Anesthesiologist ☐ ENT physician ☐ RT ☐ Other - Specify below:
____

Who will bag-mask? ☐ Resident ☐ Fellow ☐ NP ☐ Attending ☐ RT ☐ Other (Specify) ________

How will we intubate? **Method:** ☑ oral vs. ☐ nasal **ETT Type:** ☑ Cuffed ☐ Uncuffed
ETT Size: ☐ 3.0 ☑ 3.5 ☐ 4.0 ☐ 4.5 ☐ 5.0 ☐ 5.5 ☐ 6.0 ☐ 6.5 ☐ 7.0 ☐ 7.5 ☐ 8.0 ☐ Other: ____
Device: ☑ Laryngoscope ☐ LMA ____ ☐ Glidescope ____ ☐ Other: ________
Blade: ☐ Mac ____ ☐ Miller ____ ☑ Wis-Hipple 1.5
Meds: ☑ Atropine ____ mg ☐ Glycopyrrolate ____ mcg
☐ Fentanyl ____ mcg ☑ Midazolam ____ mg ☑ Ketamine ____ mg ☐ Propofol ____ mg
☑ Rocuronium ____ mg ☐ Vecuronium ____ mg
Apneic Oxygenation: (YES) / NO HFNC L/min (<1y = 5L; 1-7y = 10L; ≥8y = 15L)
Other: ________

When will we intubate? (describe the timing of airway management):
☐ Prior to procedure at: ____ ☐ Mental Status Changes ☑ Hypoxemia refractory to ~~CPAP~~ HFNC: SpO2 < ____%
☐ Ventilation failure refractory to NIV ☐ Loss of Airway Protection ☐ Other: ________

Backup? Advanced Airway Provider: ☑ Attending ☐ Anesthesia ☐ ENT ☐ Fellow ☐ Other: ________
☑ Difficult Airway Cart ☐ Difficult Airway Emergency Page ☐ Other: ________

☐ **Front page not filled out: Why?** ________

Immediate Pre-Intubation Procedure TIME OUT

Date: 5/24/2016
Time: 16:30
(Complete immediately before intubation)

☑	**Right Patient**: Confirm 2 identifiers and allergy status.<br>Did the patient eat or drink in the last 6 hours?
☑	**Right Plan**: Review and revise the FRONT PAGE plan
☑	**Right Prep**: Patient accessible and positioned correctly, bed cleared for intubation, working IV?
☑	**Right Equipment**: SOAP (e.g Suction, Oxygen, Airway, Personnel), IV fluid bolus readily available?
☑	**Right Monitoring**: BP cycling frequently, different extremity from pulse ox, pulse ox volume?
☑	**Right Rescue plan**: Difficult Airway cart/kit and equipment available? Who can we call for assistance?<br>***Double press ASCOM Alert Button, or call Emergency# 4CODE***<br>***State: "Stat Airway Emergency" (Provide Location)***
☑	**Right Attitude**: State out loud:<br>**"IF anybody has a concern at any time during the procedure please SPEAK UP."**

Other PATIENT SPECIFIC preparation:

Apneic Oxygenation -> will keep same flow (pt on HFNC) and increase FiO_2 100%.

Post-Procedure TIME OUT

☐ **All team members performed well without technical/communication challenges.**

Or

Briefly describe below (comments by provider)

	What did we do well?	What can we improve upon?
1. RT	No Desaturation	
2. Nurse	Good closed loop communication	
3. Resident		
4. NP/PA		
5. Fellow	No Desaturation	Took too long to intubate
6. Attending		

ETT Cuff adjusted to minimal leak: (YES) / NO
Goal SBS -2 **(- 3 to +2)**
Was the patient difficult to ventilate? YES/(NO)
Was the Patient difficult to Intubate? YES/(NO)

****If Yes to either question please remember to put an ALERT in Epic and a SIGN at the bedside.****

Back page completed by (PRINT): Nadkarni
Intubated by: Newton
☑ NEAR4Kids data form completed after intubation?

ver. 5

NOT PART OF THE MEDICAL RECORD

Airway Bundle Checklist (FRONT and BACK)

Date: 5/24/2016 [Place patient sticker/stamp here]
Sim, Jane
MRN: 12345678
Time: 15:00
Front page completed (check all that apply):

☐ On admission ☑ During rounds ☐ After Rounds ☐ Just prior to intubation ☐ Prior to Extubation

By Nadkarni

Assessment for ANTICIPATED Airway Management

Intubation Risk Assessment

**Difficult Airway**	**History** of difficult airway?	YES	(NO)
	**Physical**? (e.g. small mouth, small jaw, large tongue, or short neck)	YES	(NO)
**At Risk For:**	High risk for rapid **desaturation** during intubation	YES	(NO)
	(Increased ICP), pulmonary hypertension, need to avoid **hypercarbia**	(YES)	NO
	**Unstable hemodynamics** (e.g. hypovolemia, potential need for fluid bolus, vasopressor, CPR)	YES	(NO)
	Other risk factors? Avoid Hypoxemia, Hypercarbia	YES	(NO)

Planning (all risk noted above should be considered in plan)

Who will intubate? (Specify primary provider who will perform **first** laryngoscopy):

☐ Resident ☑ Fellow ☐ NP ☑ Attending ☐ Anesthesiologist ☐ ENT physician ☐ RT ☐ Other - Specify below:

Who will bag-mask? ☐ Resident ☑ Fellow ☐ NP ☑ Attending ☐ RT ☐ Other (Specify) ____

How will we intubate? **Method:** ☑ oral vs. ☐ nasal **ETT Type:** ☑ Cuffed ☐ Uncuffed

ETT Size: ☐ 3.0 ☐ 3.5 ☐ 4.0 ☐ 4.5 ☐ 5.0 ☐ 5.5 ☐ 6.0 ☐ 6.5 ☐ 7.0 ☐ 7.5 ☐ 8.0 ☐ Other: ____

Device: ☑ Laryngoscope ☐ LMA ____ ☐ Glidescope ____ ☐ Other: ____

Blade: ☐ Mac ____ ☐ Miller ____ ☑ Wis-Hipple 1.5

Meds: ☐ Atropine ____mg ☐ Glycopyrrolate ____mcg
☑ Fentanyl ____mcg ☐ Midazolam ____mg ☐ Ketamine ____mg ☑ Propofol ____mg
☑ Rocuronium ____mg ☐ Vecuronium ____mg

Apneic Oxygenation: (YES) NO 5 L/min (<1y = 5L; 1-7y = 10L; ≥8y = 15L)

Other: ____

When will we intubate? (describe the timing of airway management):

☐ Prior to procedure at: ____ ☐ Mental Status Changes ☐ Hypoxemia refractory to CPAP: SpO2 < ____%

☐ Ventilation failure refractory to NIV ☐ Loss of Airway Protection ☑ Other: Increased ICP

Backup? Advanced Airway Provider: ☑ Attending ☐ Anesthesia ☐ ENT ☐ Fellow ☐ Other: ____
☑ Difficult Airway Cart ☐ Difficult Airway Emergency Page ☐ Other: ____

☐ **Front page not filled out: Why?** ____

ver. 5

NOT PART OF THE MEDICAL RECORD

Immediate Pre-Intubation Procedure TIME OUT

Date: 5/24/2016

Time: 16:00

(Complete immediately before intubation)

- ☑ **Right Patient**: Confirm 2 identifiers and allergy status. Did the patient eat or drink in the last 6 hours?
- ☑ **Right Plan**: Review and revise the FRONT PAGE plan
- ☑ **Right Prep**: Patient accessible and positioned correctly, bed cleared for intubation, working IV?
- ☑ **Right Equipment**: SOAP (e.g Suction, Oxygen, Airway, Personnel), IV fluid bolus readily available?
- ☑ **Right Monitoring**: BP cycling frequently, different extremity from pulse ox, pulse ox volume?
- ☑ **Right Rescue plan**: Difficult Airway cart/kit and equipment available? Who can we call for assistance?
  Double press ASCOM Alert Button, or call Emergency# 4CODE
  State: "Stat Airway Emergency" (Provide Location)
- ☑ **Right Attitude**: State out loud:
  "IF anybody has a concern at any time during the procedure please SPEAK UP."

Other PATIENT SPECIFIC preparation:

Post-Procedure TIME OUT

☐ **All team members performed well without technical/communication challenges.**

Or

Briefly describe below (comments by provider)

	What did we do well?	What can we improve upon?
1. RT	No Desaturation	Mother Chose not to stay
2. Nurse	Good closed loop communication	
3. Resident		
4. NP/PA		
5. Fellow		Took longer to intubate
6. Attending		Head up 30° after intubation

ETT Cuff adjusted to minimal leak: (YES) / NO

Goal SBS -2 **(- 3 to +2)**

Was the patient difficult to ventilate? YES/(NO)

Was the Patient difficult to Intubate? YES/(NO)

If Yes to either question please remember to put an ALERT in Epic and a SIGN at the bedside.

Back page completed by (PRINT): Nadkarni

Intubated by: Newton

☑ NEAR4Kids data form completed after intubation?

ver. 5

NOT PART OF THE MEDICAL RECORD
