# Supplementary figures and images for: Effect of apneic oxygenation with intubation to reduce severe desaturation and adverse tracheal intubation-associated events in critically ill children
Source: Crit Care. 2023 Jan 17;27:26. doi: 10.1186/s13054-023-04304-0 (PMC9847056; doi:10.1186/s13054-023-04304-0)

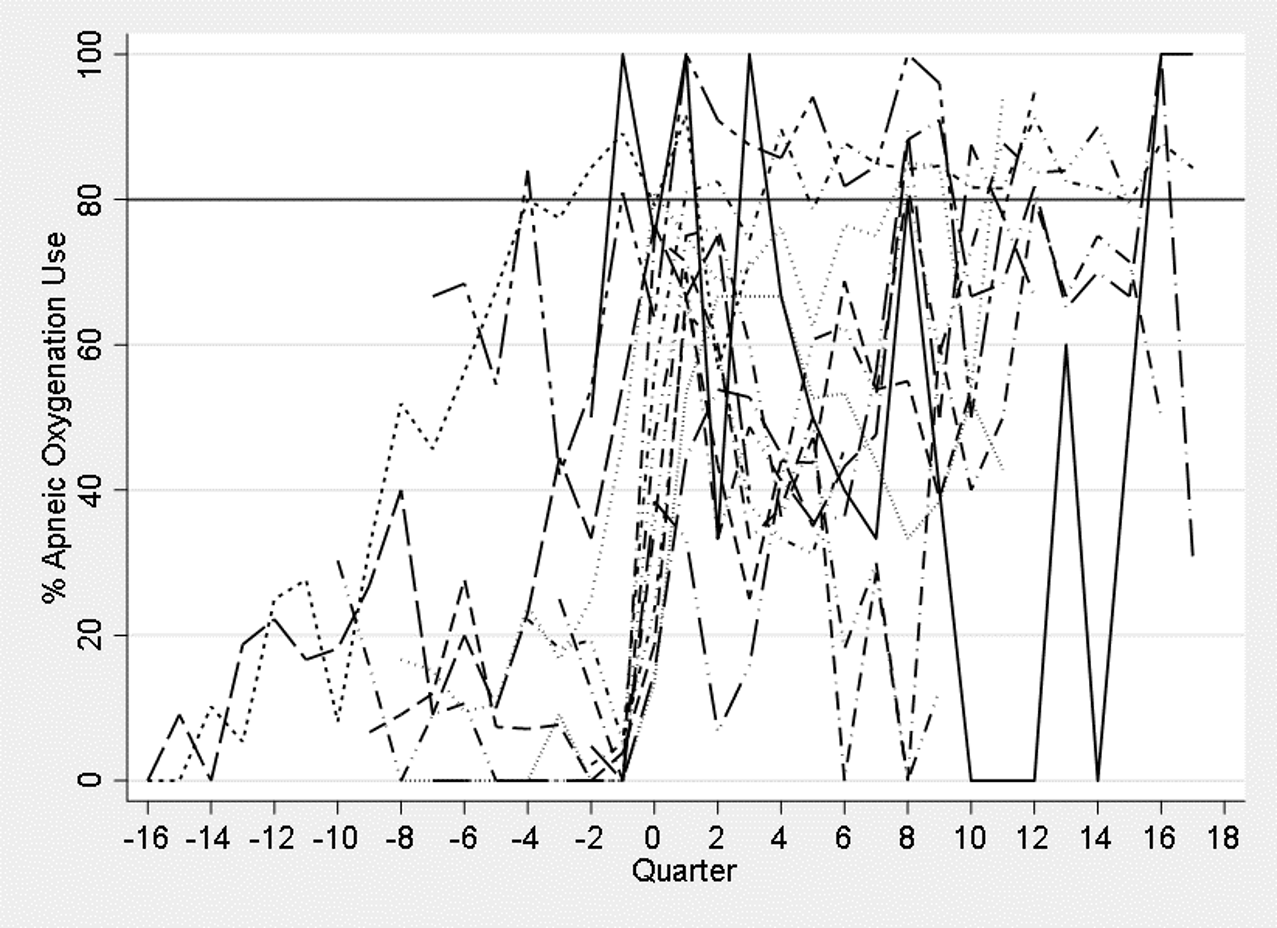

Supplement: Supplementary file 3 — Additional file 3. Supplemental Figure A. Apneic Oxygenation (AO) Use (%) before and after implementation of the intervention across the sites. [file 13054_2023_4304_MOESM3_ESM.tif]
