# Supplementary material for: Effect of apneic oxygenation with intubation to reduce severe desaturation and adverse tracheal intubation-associated events in critically ill children
Source: Crit Care. 2023 Jan 17;27:26. doi: 10.1186/s13054-023-04304-0 (PMC9847056; doi:10.1186/s13054-023-04304-0)
Supplement: Supplementary file 4 — Additional file 4. Supplemental Table B. Multivariable analysis: The Occurrence of Adverse Tracheal Intubation Associated Events in Patients who received Tracheal Intubations Before and After Apneic Oxygenation Implementation [file 13054_2023_4304_MOESM4_ESM.docx]

Supplemental Table B. Multivariable analysis: The Occurrence of Adverse Tracheal Intubation Associated Events in Patients who received Tracheal Intubations Before and After Apneic Oxygenation Implementation

| Outcome | Pre-intervention  n=2,554 | Post-intervention  n=3,995 | Unadjusted Odds Ratio (95% CI) | P-value |
| --- | --- | --- | --- | --- |
| Adverse TIAE | 324 (12.7%) | 461 (11.5%) | 0.90 (0.55-1.45) | 0.662 |
| Severe TIAE | 132 (5.2%) | 187 (4.7%) | 0.90 (0.60-1.36) | 0.619 |
| Multiple attempts | 249 (9.8%) | 336 (8.4%) | 0.85 (0.61-1.19) | 0.349 |
| Severe Hypoxemia (SpO2<80%) | 442 (17.3%) | 581 (14.5%) | 0.81 (0.61-1.09) | 0.164 |

Footnote: Unadjusted odds ratio was calculated with logistic regression with generalized estimating equations.

CI denotes confidence interval.
